# (-)-Epicatechin metabolites as a GPER ligands: a theoretical perspective

**DOI:** 10.1007/s11030-024-10968-9

**Published:** 2024-08-17

**Authors:** Rodolfo Daniel Ávila-Avilés, Erick Bahena-Culhuac, J. Manuel Hernández-Hernández

**Affiliations:** 1 Laboratory of Epigenetics of Skeletal Muscle Regeneration, Department of Genetics and Molecular Biology, Centre for Research and Advanced Studies of IPN (CINVESTAV), Mexico City, Mexico; 2 Transdisciplinary Research for Drug Discovery, Sociedad Mexicana de Epigenética y Medicina Regenerativa A. C. (SMEYMER), Mexico City, Mexico

**Keywords:** Epicatechin, Metabolites, GPER, Molecular dynamics simulation, DFT, Molecular docking

## Abstract

Diet habits and nutrition quality significantly impact health and disease. Here is delve into the intricate relationship between diet habits, nutrition quality, and their direct impact on health and homeostasis. Focusing on (-)-Epicatechin, a natural flavanol found in various foods like green tea and cocoa, known for its positive effects on cardiovascular health and diabetes prevention. The investigation encompasses the absorption, metabolism, and distribution of (-)-Epicatechin in the human body, revealing a diverse array of metabolites in the circulatory system. Notably, (-)-Epicatechin demonstrates an ability to activate nitric oxide synthase (eNOS) through the G protein-coupled estrogen receptor (GPER). While the precise role of GPER and its interaction with classical estrogen receptors (ERs) remains under scrutiny, the study employs computational methods, including density functional theory, molecular docking, and molecular dynamics simulations, to assess the physicochemical properties and binding affinities of key (-)-Epicatechin metabolites with GPER. DFT analysis revealed distinct physicochemical properties among metabolites, influencing their reactivity and stability. Rigid and flexible molecular docking demonstrated varying binding affinities, with some metabolites surpassing (-)-Epicatechin. Molecular dynamics simulations highlighted potential binding pose variations, while MMGBSA analysis provided insights into the energetics of GPER-metabolite interactions. The outcomes elucidate distinct interactions, providing insights into potential molecular mechanisms underlying the effects of (-)-Epicatechin across varied biological contexts.

**Supplementary Information:**

The online version contains supplementary material available at 10.1007/s11030-024-10968-9.

## Introduction

Diet habits and nutrition quality are directly associated with a variety of effects in health and homeostasis [1]. Therefore, it is of the utmost interest to elucidate the mechanism that the variety of food-contained components have in the organism. (-)- Epicatechin is a natural flavanol present in different foods such as green tea, cocoa, red wine, pome fruits and grapes [2–5]; and has been reported to have positive effects on the prevention of cardiovascular diseases and diabetes, due to its antioxidant and antihypertensive properties [2–4, 6–13].

Different studies oriented to understand the absorption, metabolism, distribution and excretion (ADME) of (-)-Epicatechin [14, 15] have revealed that (-)-Epicatechin is metabolized on the proximal and distal gastrointestinal tract in humans. The resulting metabolites are absorbed to be transported towards different tissues and finally, to be excreted by the urinary system.

The metabolism of (-)-Epicatechin involves a complex interplay of enzymes and biochemical processes. In the phase I metabolism, cytochrome P450 enzymes, such as CYP3A4, may be involved in the oxidation of (-)-Epicatechin. Oxidation can occur at the B-ring or the C-ring of the molecule, resulting in the formation of different metabolites[14]. In the phase II metabolism, conjugation enzymes such as UDP-glucuronosyltransferases and sulfotransferases can catalyze the conjugation of (-)-Epicatechin and its metabolites with glucuronic acid and sulfate, respectively. Conjugation with glucuronic acid and sulfate can increase the water solubility of the metabolites and facilitate their excretion in the urine[14].

Additionally, microbial enzymes in the gastrointestinal tract can play a significant role in the transformation of (-)-Epicatechin and its metabolites through microbial fermentation. Microbial fermentation can lead to the formation of metabolites such as 5-(3', 4'-dihydroxyphenyl)-*γ*-valerolactone (5-C-RFM) and 5-(3', 4'-dihydroxyphenyl)-*γ*-hydroxyvaleric acid (5-HV). These metabolites can be absorbed in the large intestine and excreted in the urine[14].

ADME studies revealed the existence of more than 20 (-)-Epicatechin metabolites on the human circulatory system, during the first 8 h after oral ingestion of 60 mg of a ^14^C-Epicatechin. These metabolites are structurally related to (-)-Epicatechin, corresponding to O-methylated, O-glucuronidated, or O-sulfated conjugates that maintain an intact flavanol ring, and C5 side chain ring fission metabolites. The most abundant (-)-Epicatechin metabolites identified are (-)-Epicatechin-3′-O-glucuronide (E3′G), (-)-Epicatechin-3′sulfate (E3′S), 3′-O-metyl-(-)-Epicatechin-5-sulfate (3′ME5S), 3′-O-metyl-(-)-Epicatechin-7-sulfate (3′ME7S), 5-(4′-hidroxyphenyl)-*γ*-verolactone-3′-sulfate (*γ*VL3′S), and 5-(4′-hidroxyphenyl)-*γ*-verolactone-3′-O-glucuronide (*γ*VL3′G) [15].

A well-documented effect of oral ingestion of (-)-Epicatechin, is the activation of the nitric oxide synthase (eNOS) [16, 17]. In addition, different protein receptors with tyrosine kinase activity that interact with G proteins have been associated to nitric oxide production. Indeed, the G protein-coupled estrogen receptor (GPER) has been identified as the target receptor for (-)-Epicatechin to induce its molecular effects [8, 18]. GPER is expressed in various tissues and cell types, including endothelial cells and has been linked to protective effects on the cardiovascular system [18]. GPER protein is structured by 7 helixes: H1 (residues 59 to 87), H2 (residues 93 to 121), H3 (residues 129 to 160), H4 (residues 165 to 195), H5 (residues 212 to 244), H6 (residues 252 to 286) and H7 (residues 300 to 326); an extracellular domain (residues 1 to 58), and an intracellular domain (residues 327 to 375)[8].

GPER has been implicated in estrogen signaling pathways across various biological systems [19]. In fact, GPER interacts with 17*β*-estradiol (E2) as an estrogen receptor, activating heterotrimeric G proteins and several effectors such as proto-oncogene tyrosine-protein kinase (Src), ERK1/2, adenylate cyclase, and sphingosine kinase (SphK), among others [20]. These pathways lead to the production of cAMP, activation of PI3K, and mobilization of intracellular calcium [21–25].

Therefore, the GPER pathway is involved in multiple tissues and physiological processes, including reproductive functions, endocrine regulation and metabolism, as well as the nervous, immune, and cardiovascular systems, neuroendocrine and cerebral processes, kidney function, and the musculoskeletal system [26, 27]. Furthermore, it has been associated with various pathophysiological conditions such as arterial hypertension, arthritis, immune diseases, breast, ovarian, and other types of cancer, diabetes mellitus, osteoporosis, proteinuric renal disease, and multiple sclerosis [27, 28].

The physiological role of GPER and its functional relation with classical ERs are still under investigation[29]. However, the interaction between (-)-Epicatechin and GPER has been shown to be energetically favorable[18]. Recently, Sarmiento et al. [30] designed and synthesized four Epi derivatives: 3-O-mesyl-(-)-epicatechin (Epi-Ms), 3-O-propargyl-(-)-epicatechin (Epi-prop), 5,7,3,4′-tetra-O-propargyl-(-)-epicatechin (Epi-4-prop), and 3,5,7,3′,4′-penta-O-propargyl-(-)-epicatechin (Epi-5-prop). The ability of these Epi derivatives to reach the GPER binding site and their ability to activate the eNOS/NO pathway was then evaluated. Epi-4-prop and Epi-5-prop showed similar activity to Epi, while Epi-prop showed better activation of the eNOS/NO pathway, compared to Epi.

Although similar effects of (-)-Epicatechin in tissue culture and in animal models have been observed, a question about the main molecular effector remains answered. Here, our aim was to evaluate the physicochemical properties of the most representative (-)-Epicatechin metabolites and its ability to bind to its recently described receptor (GPER). From this, we analyzed how the (-)-Epicatechin-derived metabolites may interact differentially with GPER, with respect to (-)-Epicatechin.

## Methods

### Physicochemical properties of (-)-Epicatechin-derived metabolites

The structure of the most representative (-)-Epicatechin and (-)-Epicatechin-derived metabolites was obtained from the PubChem database with CID numbers 72276 ((-)-Epicatechin), 76,969,982 (E3′G), 71,260,094 (E3′S), 71,579,207 (3′ME5S), 71,579,209 (3′ME7S), and 129,626,609 (*γ*VL3′S). *γ*VL3′G was generated using Gaussview Software[31].

(-)-Epicatechin and its metabolites were used to conduct a study on the electronic properties by density functional theory (DFT), in order to determine the most stable structure and the associated molecular descriptors such as molecular electrostatic potential, frontier molecular orbitals (HOMO–LUMO) and global reactivity descriptors. These included electron affinity, ionization energy, electronegativity and electrophilicity indexes, global chemical hardness and softness, and chemical potential. All DFT calculations in this study were conducted using the Gaussian 09 software suite [32] in the gas phase. The chemical model utilized the 6–311 +  + G(2d,2p) polarized basis set. For all calculations, we employed the B3LYP functional [33], which incorporates Becke’s three-parameter exchange function (B3), combined with the Lee–Yang–Parr correlation function (LYP). This method and basis set were selected due to their proven effectiveness in predicting reactivity indices of organic molecules [34].

All the vibrational analyses were evaluated in order to verify vibrational frequencies and the correspondences to potential energy surface and global minimum. Visualization of structures and orbitals of (-)-Epicatechin and its metabolites were performed using VESTA[35] and Molden[36] software. Global reactivity descriptors were evaluated using TAFF software [37] based on the following equations:

Ionization Potential (*I*) $$I = - \varepsilon_{{\text{H}}} ,$$

Electroaffinity (*A*) $$A = - \varepsilon_{L} ,$$

Chemical Potential (*μ*) $$\mu \approx \frac{1}{2}\left( {\varepsilon_{L} + \varepsilon_{H} } \right),$$

Global Hardness (*η*) $$\eta \approx \frac{1}{2}\left( {\varepsilon_{L} - \varepsilon_{H} } \right),$$

Global Softness (*S*) $$S \approx \frac{1}{2\eta } \approx \left( {\varepsilon_{L} - \varepsilon_{H} } \right)^{ - 1} ,$$

Electronegativity (*χ*) $$\chi \approx - \frac{1}{2}\left( {\varepsilon_{L} + \varepsilon_{H} } \right),$$

Electrophilicity (*ω*) $$\omega = \frac{{\mu^{2} }}{2\eta } \approx \frac{{\left( {\varepsilon_{L} + \varepsilon_{H} } \right)^{2} }}{{2\left( {\varepsilon_{L} - \varepsilon_{H} } \right)}},$$

Electron Acceptor (*ω*^+^) $$\omega^{ + } = \frac{{\left( {\varepsilon_{L} + 3\varepsilon_{H} } \right)^{2} }}{{16\left( {\varepsilon_{L} - \varepsilon_{H} } \right)}},$$

Electron Donator (*ω*^*−*^) $$\omega^{ - } = \frac{{\left( {3\varepsilon_{L} + \varepsilon_{H} } \right)^{2} }}{{16\left( {\varepsilon_{L} - \varepsilon_{H} } \right)}},$$

Net Electrophilicity (Δ*ω*^±^) $$\Delta \omega^{ \pm } = \omega^{ + } + \omega^{ - } .$$

After that, a PCA analysis was performed using global reactivity descriptors, using the software ClustVist.

### Rigid and flexible molecular docking analyses

The DFT- optimized structures of (-)-Epicatechin and metabolites were used to perform a molecular docking by Autodock Vina software 1.2.0 [38, 39]. For molecular docking, a previously simulated and validated GPER structure was used [40]. This structure was generated by GPCR I-Tasser, using the protein sequence from UniProt database (Q6FHU6). The 3D-GPER structure model was further refined through a 100 ns molecular dynamic simulation and validated using the ERRAT servers [40]. The predicted structure of GPER, was performed by GPCR I-Tasser with the protein sequence obtained from the UniProt database (Q6FHU6) [40]. After prediction the 3D-GPER structure model was further refined through a 100 ns molecular dynamic simulation and validate using ERRAT servers [40]. Docking analysis was performed using a grid box of 22.5 × 22.5 × 22.5 Å^3^ considering a 0.375 Å point spacing, the grid box was positioned in the center of the Carbon ring of Phe 206, considering previous reported analysis [8, 20, 40]. Docking analysis considered an exhaustiveness of 10, performing 10 different molecular docking analyses with a final exhaustiveness of 100.

Once a rigid molecular docking was completed, a flex molecular docking was performed for (-)-Epicatechin and metabolites. The flex molecular docking parameters considered a grid box of 22.5 × 22.5 × 26.25 Å^3^ and considering a 0.375 Å point spacing. The grid box was positioned in the center of the Carbon ring of Phe 206. Also, the flex amino acids considered that GPER structure was adjusted to Leu137, Gln138, Met141, Tyr142, Phe208, Gln215, Glu218, and Val219 following previous reported analysis[40]. The exhaustiveness was adjusted to 10, performing 10 different molecular docking analyses with a final exhaustiveness of 100.

### Molecular dynamics simulation and post-simulation analysis

After molecular docking analysis of (-)-Epicatechin and metabolites bonded to GPER, a molecular dynamics simulation was completed in order to characterize the protein:metabolite complex formation and stabilization. The best rigid molecular docking structure ranked was used as an initial structure for molecular dynamics simulation. NAMD2.13 program [41] was used to perform the molecular dynamics simulations, executing the protein-membrane tutorial (http://www.ks.uiuc.edu/Training/Tutorials/science/membrane/). The forcefields employed in the simulation was CHARMM22 [41] to describe protein and CHARMM27 [42] employed for membrane. The forcefield used for metabolites and (-)-Epicatechin was obtained from SwissParam [43] using the specific structures optimized by DFT studies. Considering that GPER is a membrane protein, we employed a membrane of 1-palmitoyl-2-oleoyl-sn-glycero-3-phosphocholine (120 × 120 Å^2^), and the system was solvated with TIP3P water model [44]. Then the neutralization of the system was adjusted to 0.15 M of NaCl and the preparation of the system was performed using VMD program [42]. After system building, it was thermalized to 310 K by Langeving dynamics, considering a 2 fs timestep.

Finally, a 100 ns simulation was carried for each system. The analysis of its trajectory was performed using VMD software. RMSD (root mean square deviation), RMSF (root mean square fluctuation) and Rg (Radius of gyration) were done with GROMACS 4.6 program[45]; all of them calculated through all time simulated. Cluster Analyses were done using GROMACS software with a 0.1 nm cutoff, and the RMSD values previously calculated. The interactions between metabolites and GPER within the most significant clusters from each analysis were visualized using the LigPlot algorithm[46]; it also was used to perform a non-covalent interaction index (NCI) analysis associated by promolecular densities (*ρ* pro), considering all atomic contributions and evaluated using NCIPlot software[47].

### MMGBSA analysis

Molecular dynamics simulation of GPER protein and (-)-Epicatechin metabolites were used to perform a MMGBSA analysis using MolAICal software [48]. Employing the three-trajectory approach, MolAICal utilizes log files generated from molecular dynamics (MD) simulations conducted via NAMD software. The MMGBSA analysis computes the binding free energy (ΔG_bind_) based on the following equations:

$$ \Delta {\text{G}}_{{{\text{bind}}}} = \Delta {\text{H}} - {\text{T}}\Delta {\text{S}} \approx \Delta {\text{E}}_{{{\text{MM}}}} + \Delta {\text{G}}_{{{\text{sol}}}} - {\text{T}}\Delta {\text{S,}} $$

$$ \Delta {\text{E}}_{{{\text{MM}}}} = \, \Delta {\text{E}}_{{{\text{internal}}}} + \, \Delta {\text{E}}_{{{\text{ele}}}} + \, \Delta_{{{\text{Evdw}}}} , $$

$$ \Delta {\text{G}}_{{{\text{sol}}}} = \, \Delta {\text{G}}_{{{\text{GB}}}} + \, \Delta {\text{G}}_{{{\text{SA}}}} , $$

where, ΔE_MM_ is the gas phase MM energy, which contains the ΔE of electrostatic (ΔE_ele_), van der Waals energy (ΔE_vdw_) and internal (bond, angle, and dihedral energies) (ΔE_internal_). ΔG_sol_ is the solvation free energy. Which is the sum of the non-electrostatic solvation component (non-polar contribution) (ΔG_SA_) and the electrostatic solvation energy (polar contribution) (ΔG_GB_) [48, 49]. TΔS is the conformational entropy, typically disregarded in MMGBSA calculations unless ligands undergo no binding-induced structural changes during the simulation [48]. By excluding ligand entropy, MolAICal not only facilitates the evaluation of binding free energy but also ensures a precise and straightforward MMGBSA computations.

#### ADMET

ADMET analyses were conducted using the third version of ADMETlab [50]. This tool allowed us to capture absorption, distribution, metabolism, excretion, and toxicity parameters. Each parameter was classified as either accepted (if the values were optimal) or rejected (if the values were not optimal or only partially accepted) [50]. The specific values are presented in Supplementary Table 1. For a more comprehensive analysis, the percentage of properties within the optimal range for each category was calculated and visualized in Fig. 11.

### QSAR analysis

Quantitative structure–activity relationship (QSAR) was calculated based on the binding energy from the MMGBSA using MolAICal software by genetic algorithm [48]. This versatile tool offers the flexibility of employing deep-learning of two distinct sets of modules for molecular descriptor calculation, PaDEL-Descriptor[51] and Mordred[52]. This should enhance a more comprehensive analysis of relevant molecular features. For each molecular descriptor dataset, two methodologies were employed for model training and validation. The first method involved partitioning the data into training and validation sets. The training set consisted of five randomly selected ligands, leaving the remaining two ligands for validation. The second approach utilized a Leave-One-Out (LOO) procedure. Due to some high values in the External Q^2^ within the first methodology, LOO models were used.

## Results and discussion

**Fig. 1:**
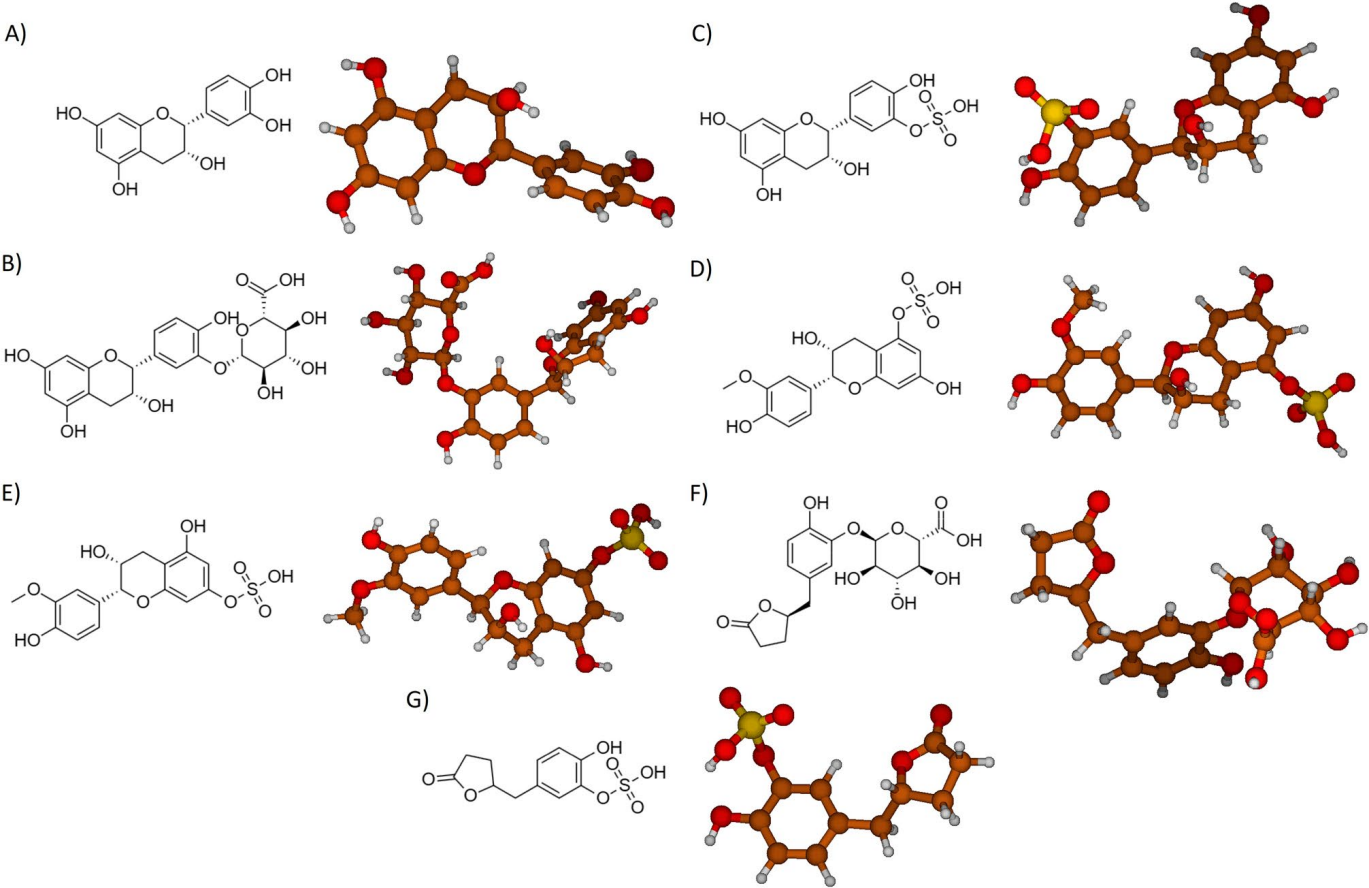
2D representation and molecular structure determination of **A** (-)-Epicatechin, and metabolites: **B** E3′G, **C** E3′S, **D** 3′ME5S, **E** 3′ME7S, **F** *γ*VL3′G, and **G** *γ*VL3′S. Code color: carbon atoms (brown), oxygen atoms (red), sulfur atoms (yellow) and hydrogen atoms (withe)

The most representative (-)-Epicatechin metabolites were studied by DFT to determine the physicochemical properties of each one. The structures obtained after energy minimization are shown in Fig. 1. (-)-Epicatechin is a flavonoid, and as such, it shares a chemical core of benzo-*γ*-pyrone that consist in two benzene rings and a dihydropyran heterocycle (named A, B and C rings). In specific, (-)-Epicatechin is a flavan-3-ol characterized by lacking the keto group at the position 4 (Fig. 1A). (-)-Epicatechin has a biotransformation process after oral intake in humans, generating different classes of metabolites; some conserve the benzo-*γ*-pyrone chemical core with O-glucuronide group addition, like (-)-Epicatechin-3′-O-glucuronide (E3′G) (Fig. 1B), or a sulfate group addition in (-)-Epicatechin-3′sulfate (E3′S) (Fig. 1C). Also, the structure accepts more than one group like 3′-O-metyl-(-)-Epicatechin-5-sulfate (3′ME5S), and 3′-O-metyl-(-)-Epicatechin-7-sulfate (3′ME7S) (Fig. 1D and E). In addition, the benzo-*γ*-pyrone chemical core could be modified to C5 side chain ring fission and form 5-(4′-hidroxyphenyl)-*γ*-verolactone-3′-O-glucuronide (*γ*VL3′G), and 5-(4′-hidroxyphenyl)-*γ*-verolactone-3′-sulfate (*γ*VL3′S) (Fig. 1F, G).

**Fig. 2:**
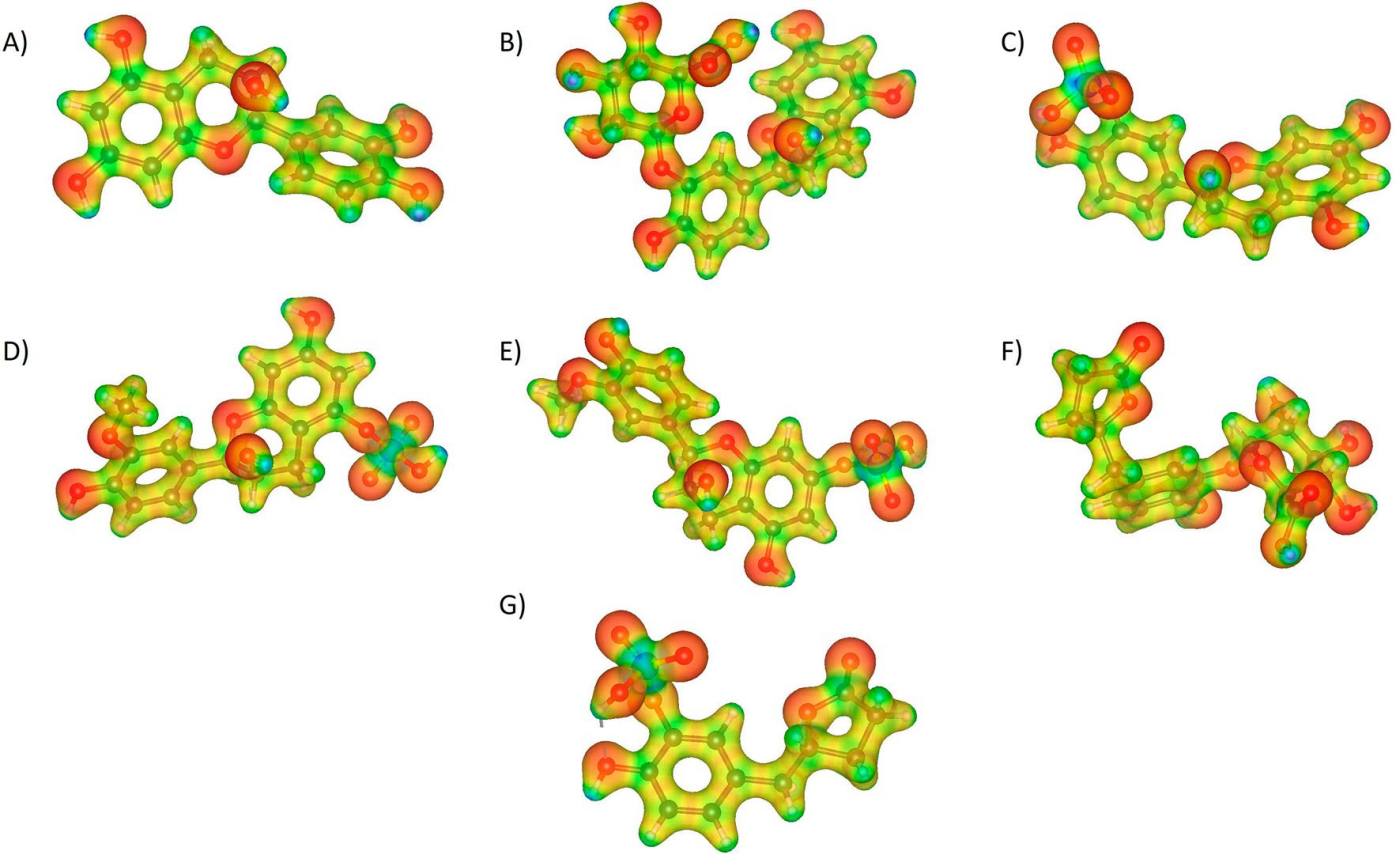
Molecular electrostatic potential representation of **A** (-)-Epicatechin, and metabolites: **B** E3′G, **C** E3′S, **D** 3′ME5S, **E** 3′ME7S, **F** *γ*VL3′G, and **G** *γ*VL3′S

A molecular potential analysis was applied to (-)-Epicatechin and metabolites in order to describe the possible electron density distribution (Fig. 2). Molecular electrostatic potential (MEP) is a descriptor that allow the prediction of molecular sites that may participate on non-covalent interactions [53]. The molecular sites with disposition of electrophilic attacks represented by blue surface are positioned in hydrogen atoms; in contrast, red surface represent molecular sites that could be part of nucleophilic attack and is located on oxygen atoms. In specific, sulfate group on E3′S, 3′ME5S, 3′ME7S, and *γ*VL3′S could induce a more characteristic reactivity to these metabolites whereas a glucuronide group on E3′G, and *γ*VL3′G suggest that these metabolites have more -OH reactive groups that could participate in more H-bonds formation.

**Fig. 3:**
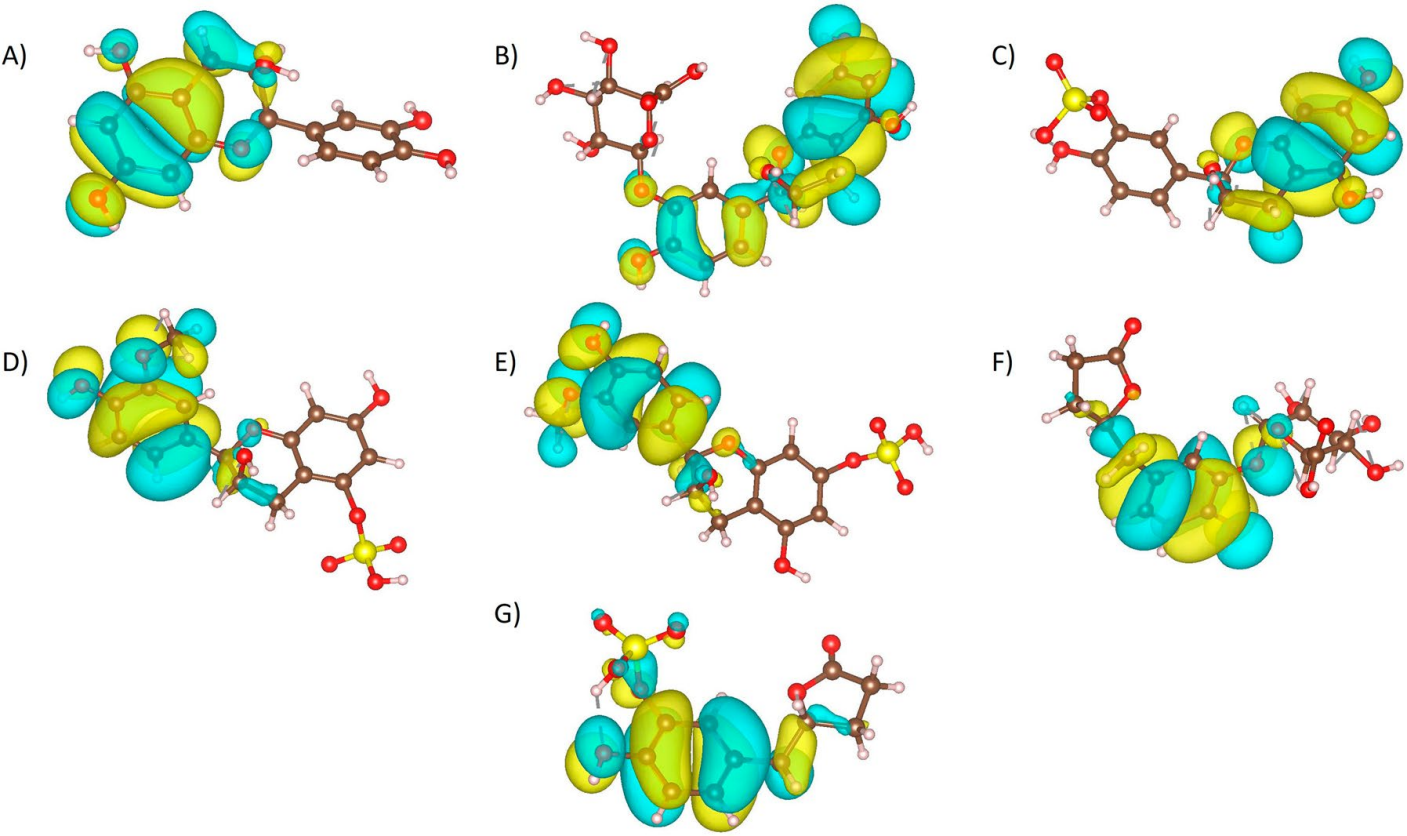
Molecular orbital HOMO representation of **A** (-)-Epicatechin, and metabolites: **B** E3′G, **C** E3′S, **D** 3′ME5S, **E** 3′ME7S, **F** *γ*VL3′G, and **G** *γ*VL3′S

Frontier molecular orbital (FMO) was calculated for metabolites and (-)-Epicatechin in order to determine the electron occupation throughout the molecule, and to predict the molecular sites that could participate donating or accepting electrons on a reaction [54]. FMO is configured by the highest occupied molecular orbital (HOMO) that reveals the tendency to donate electrons and the lowest unoccupied molecular orbital (LUMO), with the ability to accept electrons [54]. HOMO orbitals (Fig. 3) were located with more frequency on A and B rings in the benzo-*γ*-pyrone chemical core of the (-)-Epicatechin and metabolites. HOMO energy values were between − 134 kcal/mol to − 142 kcal/mol for (-)-Epicatechin and metabolites, except for *γ*VL3′S, where the homo value was -161.897 kcal/mol and the HOMO orbital was located on sulphate group (Fig. 5A). This shows that the C5 side chain ring fission of *γ*VL3′S, induces significant alteration in physicochemical properties with respect to (-)-Epicatechin.

**Fig. 4:**
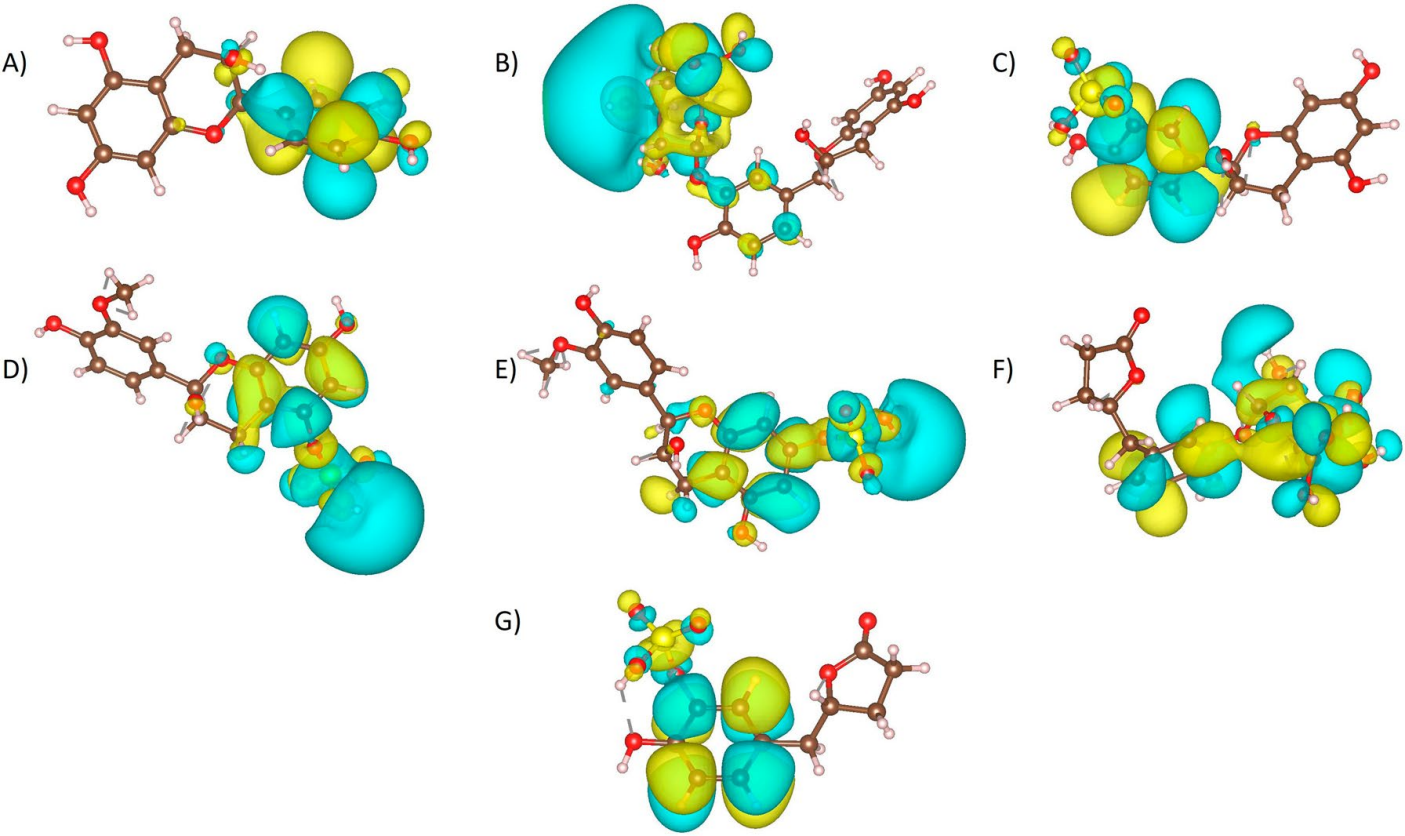
Molecular orbital LUMO representation of **A** (-)-Epicatechin, and metabolites: **B** E3′G, **C** E3′S, **D** 3′ME5S, **E** 3′ME7S, **F** *γ*VL3′G, and **G** *γ*VL3′S

**Fig. 5:**
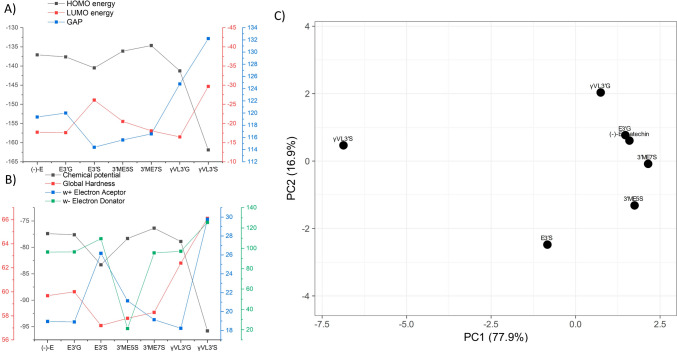
Reactivity indexes determined for (-)-Epicatechin, and metabolites: E3’G, E3’S, 3’ME5S, 3’ME7S, *γ*VL3’G, and *γ*VL3’S. **A** By HUMO, LUMO and GAP . **B** by Electrochemical properties. **C** PCA analysis of (-)- Epicatechin and metabolites according to reactivity indexes analyzed

LUMO molecular orbitals representations are presented in Fig. 4 and values are plotted on Fig. 5. LUMO orbitals were located on the C-ring of the benzo-*γ*-pyrone chemical core of the flavanols. Higher values of LUMO are predicted for metabolites with sulfate group such as E3′S, 3′ME5S, 3′ME7S, and *γ*VL3′S, suggesting that sulfate group induce on metabolites a predisposition to accept electrons. The energy difference between the HOMO and LUMO (GAP energy of HOMO–LUMO (E_LUMO-HOMO_)) can be used to determine kinetic stability and chemical reactivity of a molecule. Hence, a short gap suggests low kinetic stability and high chemical reactivity [55, 56]. The lowest GAP energy (Fig. 5A) was calculated for E3′S (114.39 kcal/mol) and the highest for *γ*VL3′S (132.21 kcal/mol), also lower GAP energies were calculated for 3′ME5S and 3′ME7S compared to (-)-Epicatechin, suggesting that a sulfate group may increase the reactivity of the metabolites that could be favorable for their interaction with GPER. In contrast, the glucuronide group on E3′G and γVL3′G have higher GAP energy values, suggesting that glucuronide increase the stability of the metabolites. Then, this higher kinetic stability is associated with a more stable interaction when the molecule associates with specific proteins.

Then, reactivity indexes were calculated for (-)-Epicatechin and metabolites (Fig. 5 and Table 1). We observed that reactivity calculated by GAP energy has a correlation with the physicochemical properties calculated, specifically, Global Hardness which measures the degree that a certain molecule is unable to perform a charge transfer. Lower values of global hardness were calculated for those metabolites with a sulfate group such as E3′S, 3′ME5S, and 3′ME7S and were compared to (-)-Epicatechin, showing that these metabolites have higher ability to transfer charge, suggesting a higher chemical reactivity and lowest kinetic stability.

**Table 1:** Reactivity indexes were calculated for (-)-Epicatechin and metabolites

Molecular descriptor/molecule	(-)-Epicatechin	E3′G	E3′S	3′ME5S	3′ME7S	*γ*VL3′G	*γ*VL3′S
HOMO energy	− 137.081	− 137.625	− 140.496	− 136.123	− 134.664	− 141.277	− 161.897
LUMO energy	− 17.724	− 17.623	− 26.105	− 20.535	− 18.091	− 16.502	− 29.681
GAP	119.357	120.001	114.391	115.587	116.573	124.775	132.216
Ionization potential	137.081	137.625	140.496	136.123	134.664	141.277	161.897
Electroaffinity	17.724	17.623	26.105	20.535	18.091	16.502	29.681
Chemical potential	− 77.403	− 77.624	− 83.301	− 78.329	− 76.377	− 78.889	− 95.789
Global hardness	59.678	60.001	57.195	57.794	58.286	62.387	66.108
Global softness	3298.988	3281.268	3442.212	3406.573	3377.768	3155.741	2978.126
Electronegativity	77.403	77.624	83.301	78.329	76.377	78.889	95.789
Electrophilicity index (*w*)	50.196	50.212	60.661	53.080	50.042	49.878	69.398
w + Electron aceptor	18.954	18.900	26.160	21.140	19.139	18.232	29.767
w − Electron donator	96.357	96.524	109.460	21.140	95.516	97.121	125.556
Electrophilicity net	115.311	115.424	135.620	120.609	114.655	115.353	155.323

All the values are in Kcal/mol

The chemical potential serves as an indicator of how likely electrons in a molecule are to transfer to other molecules[57–59]. A higher chemical potential implies greater reactivity. This descriptor was favorable for E3′S and showed acceptable values for other metabolites (see Fig. 5B). In contrast, *γ*VL3′S had the highest GAP energy and chemical potential among all the metabolites, along with significantly different values for other descriptors. This suggests that the chemical similarity between (-)-Epicatechin and *γ*VL3′S is low, suggesting a reduced ability to interact with GPER.

Another set of physicochemical descriptors that can hint at a molecule's ability to interact in a complex are electron donor and acceptor properties. In this context, E3′S exhibited the highest values for both electron donation and acceptance (as shown in Fig. 5B). Additionally, 3′ME5S is noteworthy because it has a lower electron donor index but a high electron acceptor index when compared to (-)-Epicatechin and 3′ME7S (Fig. 5B). This observation, along with lower GAP and Hardness values, and higher chemical potential, suggests that 3′ME5S possesses characteristics that could enhance its interactions with GPER.

Regarding E3′G, its chemical properties align closely with those of (-)-Epicatechin in terms of chemical potential, electron donor, and acceptor indexes. However, it exhibits an increased GAP energy (Fig. 5), implying that E3′G might have a similar interaction with GPER but potentially enhances the stability of the interaction.

A PCA analysis of metabolites using molecular descriptor information suggest that the E3′G metabolite exhibit the closest physicochemical descriptors to (-)-Epicatechin, in contrast, *γ*VL3′S has the lowest chemical similarity to (-)-Epicatechin (Fig. 5C and Table 1).

**Fig. 6:**
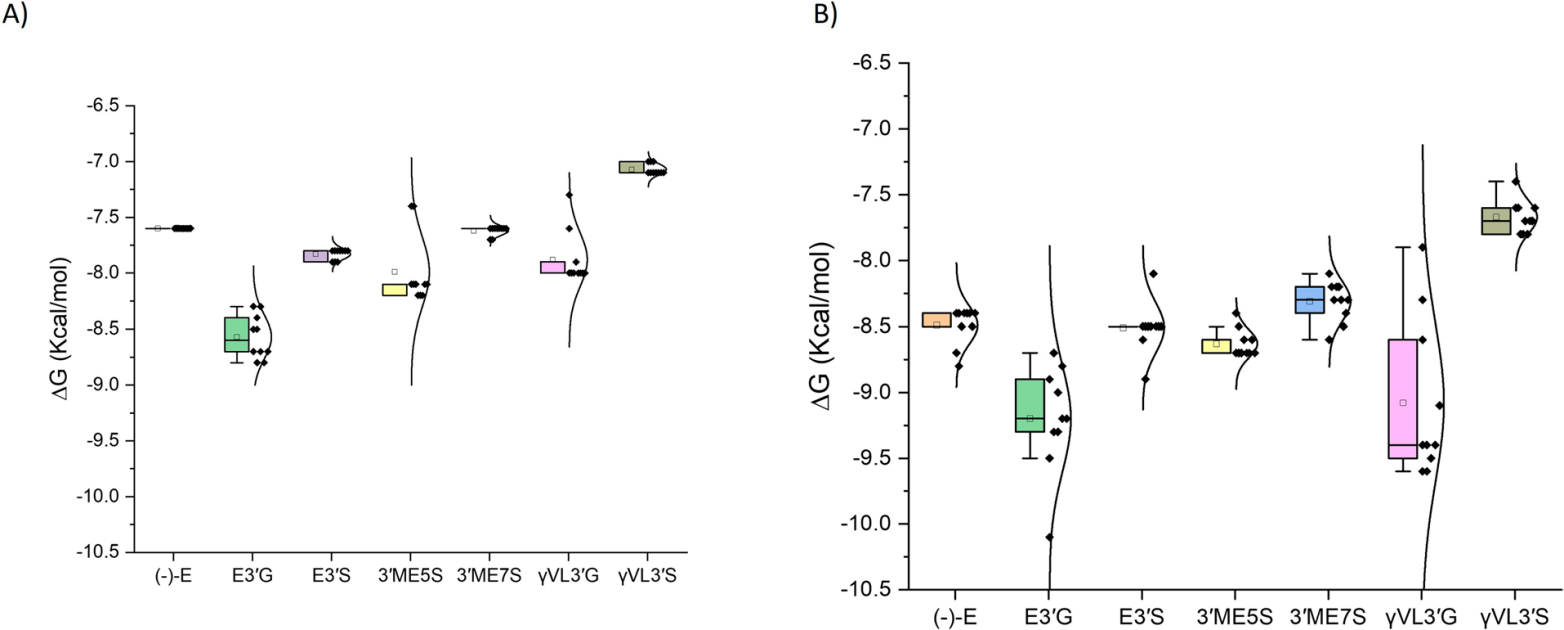
Rigid (**A**), and Flex (**B**) molecular docking of (-)-Epicatechin, and metabolites; E3′G, E3′S, 3′ME5S, 3′ME7S, *γ*VL3′G, and *γ*VL3′S with GPER receptor (plotted with Origin 8.0 software)

After DFT characterization of (-)-Epicatechin metabolites, we evaluated its binding affinity to GPER protein. To address this, we first employed rigid molecular docking (Fig. 6A). (-)-Epicatechin and metabolites exhibited favorable binding energies between − 7.0 to − 9.0 kcal/mol, in fact, (-)-Epicatechin metabolites showed better binding affinity prediction to GPER than (-)-Epicatechin. E3′G exhibit the best affinity to GPER in contrast to *γ*VL3′S, that was predicted to have less binding affinity. Then, we performed a flex molecular docking (Fig. 6B), and the results showed binding affinity energies between − 7.5 kcal/mol to − 9.5 kcal/mol. Here *γ*VL3′G and E3′G have the most favorable binding affinities. This suggests that glucuronide group induce better affinity because it has better LUMO energies associated to greater number of OH groups and a grater polarity (Fig. 5A). We observed variations on how strongly 3′ME5S and 3′ME7S bind. These molecules have the same atoms, but their sulfate group is in different positions, resulting in distinct physicochemical properties based on DFT calculations (e.g., GAP energies and electron donator index). These properties increase 3′ME5S binding affinity compared to 3′ME7S. Furthermore, *γ*VL3′S, which differs significantly in physicochemical characteristics from (-)-Epicatechin, demonstrates a lower binding affinity to GPER compared to other metabolites that have a closer chemical resemblance to (-)-Epicatechin.

**Fig. 7:**
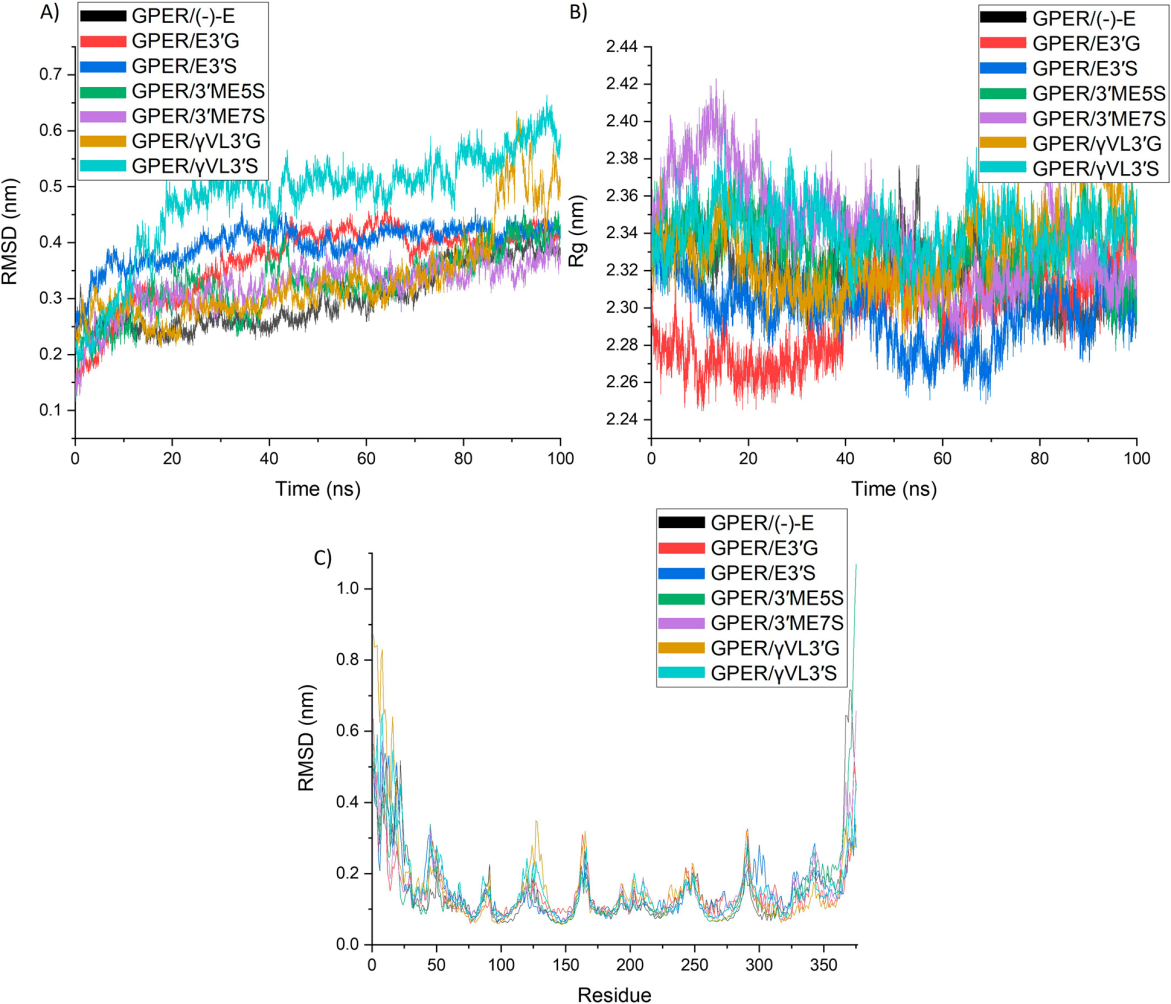
**A** RMSD, **B** Rg, and **C** RMSF of GPER protein in complex with (-)-Epicatechin, and metabolites: E3′G, E3′S, 3′ME5S, 3′ME7S, *γ*VL3′G, and *γ*VL3′S (plotted with Origin 8.0 software)

To further study the possible interaction of (-)-Epicatechin and its metabolites with GPER, we performed a 100 ns molecular dynamics simulation of GPER in a membranal context. First, we evaluated the distance between the atoms, considering the structure obtained during the time of simulation by root mean square deviation (RMSD). GPER protein RMSD in all complexes (Figure S1A) exhibited stabilization around 2.5 nm. In contrast, the RMSDs for of (-)-Epicatechin metabolites take different configurations during the simulation, compared with the stability of (-)-of Epicatechin (Figure S1B). Figure 7-A shows the RMSD value considering the alpha-carbon of the protein and the specific ligand (named complex). Here the complex stability exhibit differences depending on the metabolite; GPER/(-)-Epicatechin showed the lowest RMSD values and GPER/*γ*VL3′S the highest; GPER/γVL3′G remains stable during the simulation but the last 20 ns turn to unstable interactions. The other complex metabolites (E3′G, E3′S, 3′ME5S, and 3′ME7S) with GPER maintains stable during the simulation around 0.3 nm. RMSD analysis suggest that in contrast to (-)-Epicatechin, the metabolites could turn into different binding poses while interacting with GPER.

The molecular dynamics simulation was also evaluated in terms of radius of gyrate (Rg). Here we evaluated the fluctuation of the atoms relative to the center of mass during the simulation; Rg values give information about the compactness and stability of the structure during the simulation. Rg values of GPER protein (Figure S1C) showed lower fluctuation (0.017 nm average of standard deviation Rg values) during the simulation and that compactness was replicated on Rg values calculated for GPER complexes (0.019 nm average of standard deviation Rg values) (Fig. 7B).

The average of Rg values for GPER/metabolites were 2.322 nm; with average standard deviation of 0.019 nm. The lowest values were obtained for GPER/E3′G (2.29611 ± 0.0223 nm) and the more stable values were observed for GPER/(-)-Epicatechin. In contrast to GPER Rg (0.0189 nm standard deviation of average Rg values) and complex Rg values (0.0158 nm standard deviation of average Rg values), the calculation of Rg ligand showed differences between metabolites (0.0395 nm standard deviation of average Rg values). Here the more stable values were associated to (-)-Epicatechin (0.372 ± 0.0029 nm) and 3′ME5S (0.430 ± 0.0065 nm), and the highest values for E3′G (0.482 ± 0.0156 nm). Rg values of metabolites suggest different binding poses associated to each metabolite.

Root means square fluctuation (RMSF) was calculated during the molecular dynamic simulation (Fig. 7C). By doing this, it is possible to determine the flexibility associated to any specific residue by analysing its movement during the simulation. The flexibility increased on the intracellular domain for GPER/3′ME5S and GPER/(-)-Epicatechin. This region is reported to be important for GPER activation [8]. Higher flexibility was observed for GPER/*γ*VL3′G, GPER/*γ*VL3′S on H2, and GPER/E3′S on H7. Interestingly, these are also regions reported to be important for GPER activation [8].

Molecular dynamics simulations were used to analyse the structure of GPER and (-)-Epicatechin metabolites. Based on RMSD and Rg values, it was observed that (-)-Epicatechin metabolites have multiple binding sites with the GPER protein, suggesting that these metabolites bind slightly differently compared to the predicted binding of (-)-Epicatechin.

**Fig. 8:**
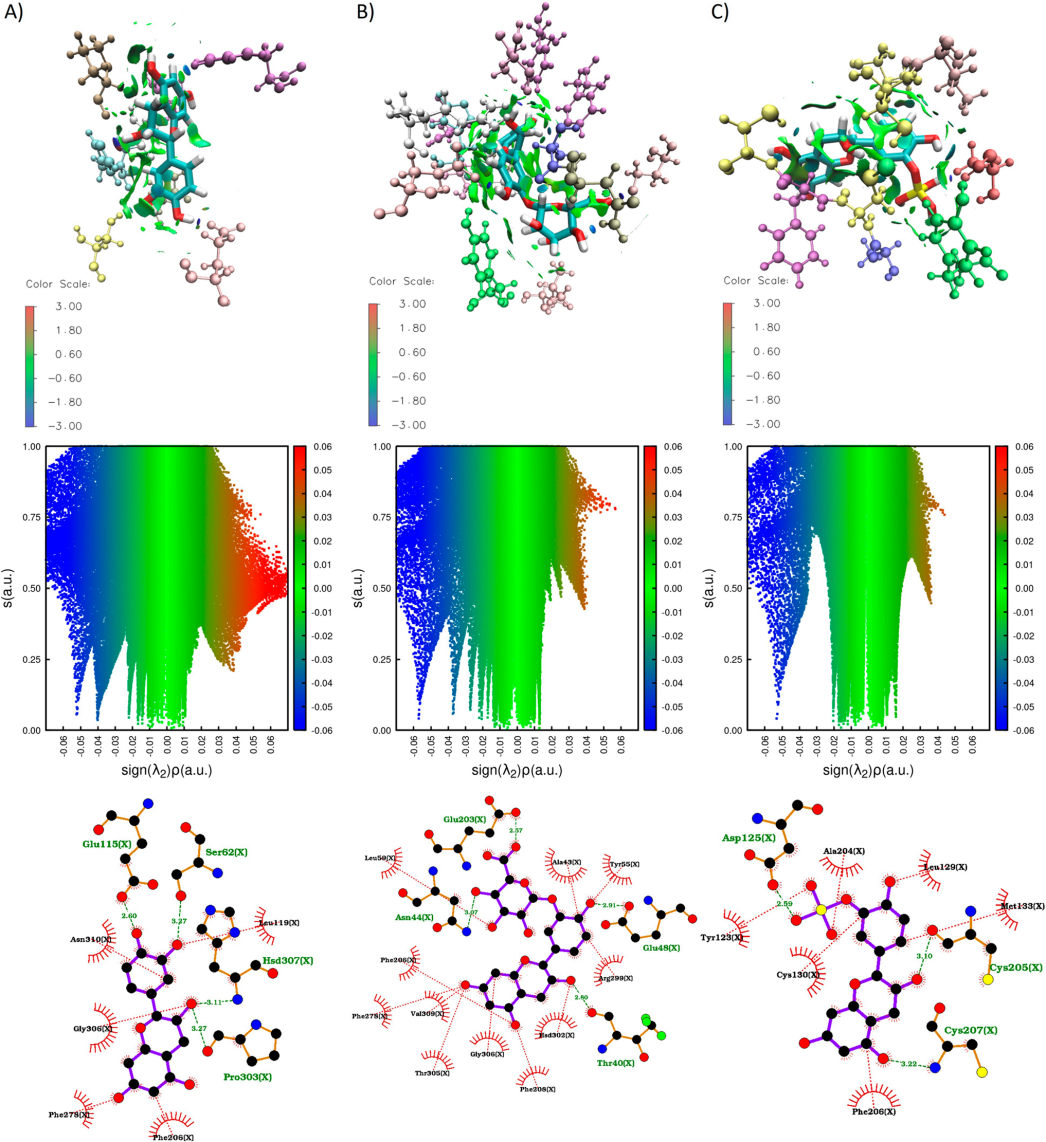
Non covalent interactions (NCI) plot (plotted with VMD 1.9.3 software) and 2D interactions (plotted with Ligplot 2.2 software) plot of complexes GPER/(-)-Epicatechin, GPER/E3′G, and GPER/E3′S. RDG vs sign (*λ*2)*ρ* plots and RDG isosurfaces (plotted with NCI 4.0 software). A color code based on sign (*λ*2)*ρ* was used: − 0.6 (blue) < 0.0 (green) < 0.7 (red)

**Fig. 9:**
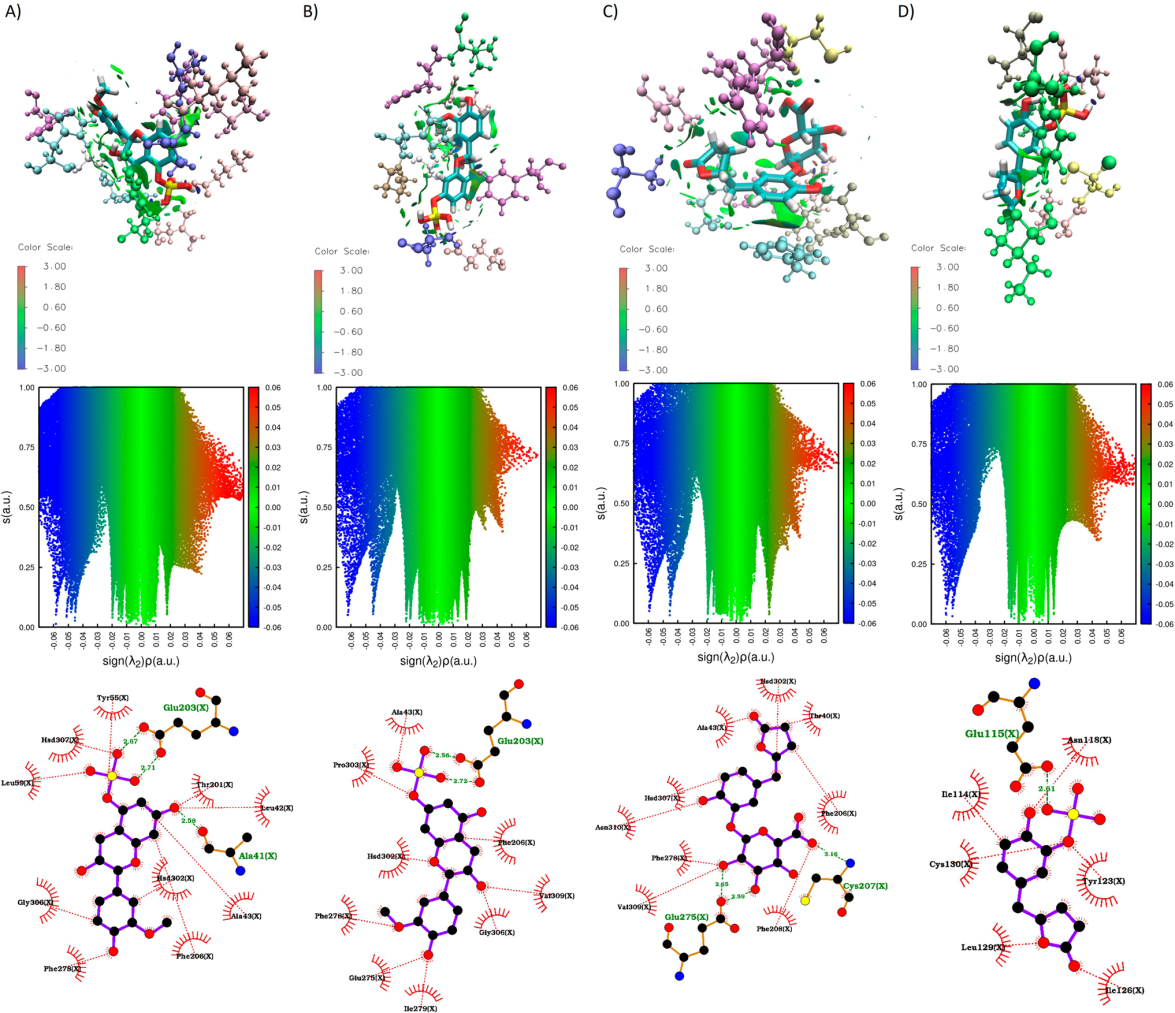
Non covalent interactions (NCI) plot (plotted with VMD 1.9.3 software) and 2D interactions plot (plotted with Ligplot 2.2 software) of complexes GPER/3′ME5S, GPER/3′ME7S, GPER/*γ*VL3′G, and GPER/*γ*VL3′S. RDG vs sign (*λ*2)*ρ* plots and RDG isosurfaces (plotted with NCI 4.0 software). A color code based on sign (*λ*2)*ρ* was used: − 0.6 (blue) < 0.0 (green) < 0.7 (red)

The complexes underwent a clustering analysis to search for the most stable structure for each complex. The results indicate that the complexes exhibit a deviation ranging from 0.1 to 0.6 nm (Figure S2). The most populated GPER/metabolite cluster was studied using NCI software to identify and describe non-covalent interactions responsible for stabilizing the binding. To characterize the non-covalent interaction between metabolites and GPER protein a sign (*λ*2)*ρ* determination was used. These interactions encompass both favorable and unfavorable interactions. To distinguish between them, the sign of the second density Hessian eigenvalue (*λ*2) multiplied by the density was employed (sign (*λ*2)*ρ*). This value effectively characterizes the strength of interactions based on density and their nature determined by the sign of the second eigenvalue. Attractive and repulsive interactions are recognized in regions where *λ*2 < 0 and *λ*2 > 0, respectively. Weak van der Waals interactions are indicated by *λ*2 ≈ 0. To visualize these regions, a color code is often used based on sign (*λ*2)*ρ*: blue for strong attractive interactions (*λ*2 < 0), green for weak van der Waals interactions (*λ* ≈ 0), and red for strong repulsive interactions (λ2 > 0) [47]. Additionally, LigPlot software was used to create 2D representations (see Figs. 8 and 9). In the case of the GPER/(-)-Epicatechin complex, it was found to be stabilized by four hydrogen bonds with Glu115, Ser62, His307, and Pro303, as well as hydrophobic interactions involving Asn310, Leu119, Gly306, Phe278, and Phe206.

The complex GPER/E3′G is bound by 4 H–Bonds interactions with Thr40, Asn44, Glu203 and Glu48, and by hydrophobic interactions with Phe206, Phe278, Val309, Thr305, Gly306, Phe208, His302, Arg299, Tyr55, Ala43 and Leu59. The H–bonds with Asn44 and Glu203 were facilitated by a glucuronide group, where the LUMO determination suggests the ability to this molecule to accept electrons. Also, interactions with the phenylalanines 206, 208, and 278 are recognized interactions [60] that promote *π*-*π* interaction that is key on the aromatic cavity of GPER [20]. The complex of GPER/E3′S is stabilized by 3 H-bonds with Asp125, Cys205 and Cys207, also by hydrophobic interactions with Cys130, Tyr123, Ala204, Leu129, Met133, Phe206. The sulfate group in contrast with glucuronide group in E3′G only form the only H–bond with Asp125, but this binding allows the formation of two H–bonds with cysteines 207 and 205. These residues, Cys 130 and 207, are reported to generate a disulphide bond and this bond participate on structural changes that induce a rearrangement in the intracellular domain associated to GPER agonist properties[60].

Moreover, the interaction between 3′ME5S and GPER is stabilized through three hydrogen bonds. Two of these bonds facilitate the sulfate group's interaction with Glu203, while the third forms a hydrogen bond with Ala41. Additionally, the complex is reinforced by hydrophobic interactions involving Tyr55, His307, Leu59, Gly306, Phe278, His302, Phe206, Ala43, Thr201, and Leu42. The dual hydrogen bond with Glu203 contributes significantly to the overall stability of the complex and concurrently promotes π-π interactions with Phe206 and Phe278. The complex of GPER/3′ME7S like 3′ME5S maintains the double H-bond with Glu203 and sulfate group, but the H-bond with Ala41 is not reported. This metabolite is also stabilized by interactions with Phe206, Val309, Gly306, Ile279, Glu275, Phe278, His302, Pro303, and Ala43. The *γ*VL3′G metabolite stabilizes its binding with GPER through 2 H-bonds by the glucuronide group with Cys207 and Glu275; and by hydrophobic interactions with Phe208, Phe206, Thr40, His302, Ala43, His307, Asn310, Phe278 and Val309. The glucuronide group participates on important interactions with GPER by the presence of C5 side chain ring fission on the characteristic benzo-γ-pyrone chemical core. Finally, the complex of GPER/γVL3′S is stabilized by hydrophobic interactions with Asn118, Tyr123, Ile126, Leu129, Cys130, and Ile114; and by an H-bond with Glu115.

**Fig. 10:**
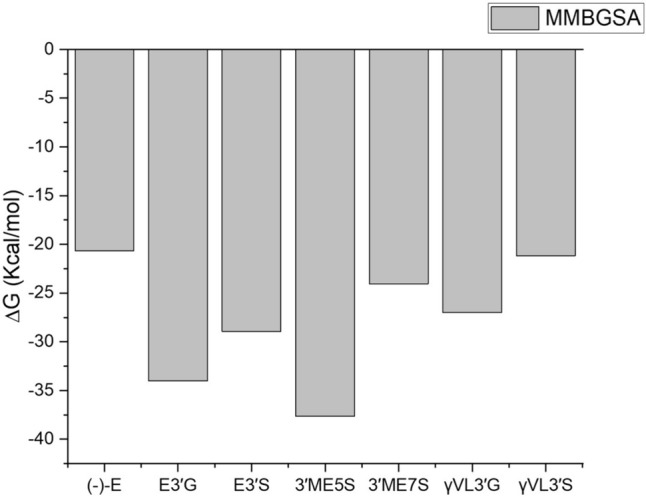
Binding energy calculated for MMGBSA for GPER protein in complex with (-)-Epicatechin, and metabolites: E3′G, E3′S, 3′ME5S, 3′ME7S, *γ*VL3′G, and *γ*VL3′S (plotted with Origin 8.0 software)

We also evaluated the GPER binding properties of (-)-Epicatechin metabolites by MMBGSA analysis. All the (-)-Epicatechin metabolites exhibit better binding affinity to GPER in contrast to (-)-Epicatechin (Fig. 10). Among the metabolites considered, 3′ME5S stands out with the highest binding affinity at − 37.61 kcal/mol. This superior affinity is correlated with a more extensive array of non-covalent interactions, as evident from the sign(*λ*2)*ρ* distribution illustrated in Fig. 9A. Specifically, the distribution pattern is linked to the formation of a double hydrogen bond in the sulfate group. Furthermore, the non-covalent interactions surrounding van der Waals energies are evenly dispersed around the molecule, as depicted in the isosurface of Fig. 9A. In contrast, E3′G, E3′S, and 3′ME7S display binding energies of − 34.02, − 28.95, and − 24.06 kcal/mol, respectively. The sign (*λ*2)*ρ* plot for these metabolites indicates a reduction in density (depicted by red dots) of repulsive interactions with positive values of sign(*λ*2)*ρ* (refer to plots in Figs. 8B, C, and 9B), as compared to the (-)-Epicatechin plot. In that case the glucuronide and sulfate groups enhance the formation of non-covalent interactions in van der Waals energies (see isosurfaces plotted on Figs. 8B, C, and 9B) where the interaction as enhanced showing by the isosurfaces distribution around the metabolites.

In *γ*VL3′G, the H–Bonds interactions on the glucuronide group participates on attractive interactions and enhance van der Waals interactions (see Fig. 9C) with GPER; in comparation to (-)-Epicatechin, that shows a binding affinity energy of − 26.98 kcal/mol. Finally, *γ*VL3′S complex exhibit a binding affinity of − 21.17 kcal/mol; this is probably associated to attractive interactions where the H-bond formation induce a sign(*λ*2)*ρ* units around -0.06 relative to other metabolites.

The efficacy of (-)-Epicatechin and its metabolites can be significantly influenced by their ADMET properties: absorption, distribution, metabolism, excretion, and toxicity. For decades, ADMET analysis has been a critical consideration in drug development, determining whether a drug molecule reaches its target protein and how long it remains in the bloodstream [50, 61]. Furthermore, studies have shown that ADMET properties can change during metabolism, affecting the drug's overall effectiveness [62]. Therefore, understanding these properties is essential for predicting the pharmacokinetic behavior and potential success of (-)-Epicatechin and its metabolites. Advances in technology have provided numerous in silico options for analyzing these properties [61]. In this study, we utilized ADMETlab in its third version [50], a web-based service that offers detailed analysis of various properties within each aspect of the ADMET profile. This tool not only provides specific values but also indicates whether these values fall within the optimal range, enhancing our understanding of the drug's potential efficacy [50].

**Fig. 11:**
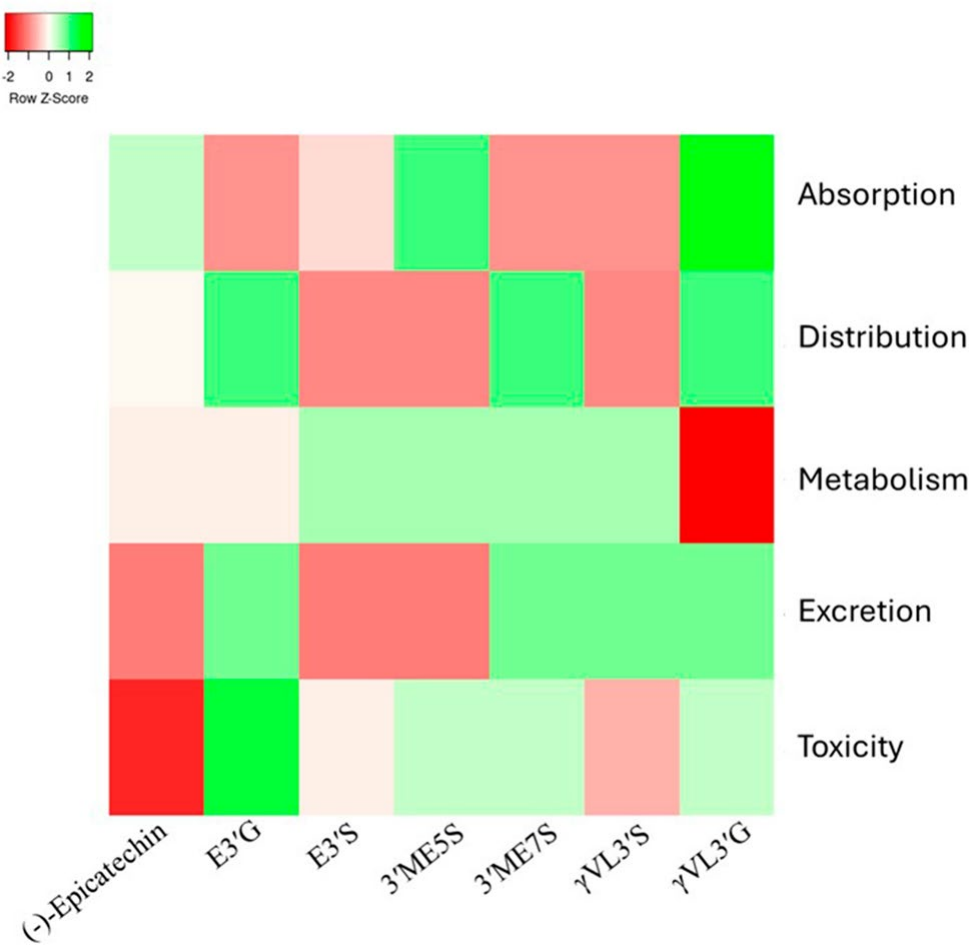
Heatmap showing the percentage of molecular properties within the optimal range for absorption, distribution, metabolism, excretion, and toxicity. Red indicates poor performance, while green indicates good performance

In terms of absorption, two metabolites outperformed (-)-Epicatechin (33.33%) in the percentage of properties within the optimal range: 3′ME5S (44.44%) and *γ*VL3′G (55.55%) (Fig. 11). Notably, 3′ME5S was the only metabolite with optimal results in the Parallel Artificial Membrane Permeability Assay, indicating a high likelihood of efficient membrane crossing. Conversely, *γ*VL3′G was the only metabolite to show positive results in bioavailability categories: F-20% and F-30%. Regarding distribution, three metabolites: E3′G, 3′ME7S, and *γ*VL3′G showed a better property profile than (-)-Epicatechin (50%), with each achieving a 62.5% optimal range (Fig. 11). These metabolites demonstrated superior performance in Plasma Protein Binding and did not inhibit Organic Anion Transporting Polypeptide 1B1 (OATP1B1), suggesting they are less likely to interfere with OATP1B1-mediated uptake and the efficacy will not be damaged by plasma protein binding.

In terms of metabolism, most metabolites showed a higher percentage of optimal properties, which is expected given their already metabolized status. Specifically, E3′S, 3′ME5S, 3′ME7S, and *γ*VL3′S were identified as substrates for CYP2C9 but did not act as substrates or inhibitors for other CYP enzymes. For excretion, E3′G, 3′ME7S, *γ*VL3′S, and *γ*VL3′G demonstrated superior clearance rates, which is likely to enhance the efficiency of these metabolites. Finally, in terms of toxicity, E3′G had the highest percentage of optimal values at 65%, followed by *γ*VL3′G, 3′ME7S, and 3′ME5S, each with 55%. E3′S and *γ*VL3′S showed 50% and 45% optimal values, respectively, while (-)-Epicatechin had the lowest at 35%. Thus, these metabolites are less likely to cause toxicity. Overall, the metabolism of (-)-Epicatechin produces metabolites with improved ADMET profiles, which could enhance the therapeutic use of (-)-Epicatechin. Among these, *γ*VL3′G stands out with the best overall results in the screening.

To gain inside of relevant molecular descriptors to improve the GPER interaction based on (-)-Epicatechin or metabolites interactions a QSAR methodology was applied. Within (-)-Epicatechin and metabolites as a set, there were 431 3D-descriptors and 1,444 2D-descriptors calculated with PaDEL, while 1665 descriptors with Mordred. For the QSAR analysis, we selected the regression model with the best adjusted *R*^2^ for each molecular descriptor dataset to ensure robustness and accuracy. Remarkably all the models showed high adjusted *R*^2^, indicating a high level of predictive accuracy. The optimal regression model for Mordred had an adjusted *R*^2^ of 0.9998 (Figure S3A):

Model 1: MMGBSA: 416.81467 + (− 205.30923) * RNCG + (− 54.65308) * MWC05 + (2.71864) * ATSC6p.

This model integrates the Relative Negative Charge (RNCG), Walk Count of Leg-5 (MWC05), and Centered Moreau-Broto Autocorrelation of Lag 6 weighted by Pauling EN (ATSC6p) as pivotal molecular descriptors. As RNCG is calculated by taking the charge of the most negative atom and dividing it by the total negative charge, this could suggest that a wider distribution of negative charge could enhance the binding of molecules within the system[63]. PaDEL 3D showcased an adjusted *R*^2^ of 0.9970 in its most effective model (Figure S3B):

Model 2: MMGBSA: − 19.18640 + (− 0.00494) * TDB10m + (− 7.04049) * RDF105p + (1.64234) * RDF115e.

This model encapsulates 3D topological distance-based autocorrelation—lag 10/weighted by mass (TDB10m), Radial distribution function—105/weighted by relative polarizabilities (RDF105p), and by relative Sanderson electronegativities (RDF115e) as essential descriptors. This model relies on the spatial arrangement of the ligand’s features. PaDEL 2D, demonstrated the highest adjusted *R*^2^ of 0.9999 for its optimal model (Figure S3C):

Model 3: MMGBSA: − 10.90223 + (− 0.04025) * ATS7s + (1.52915) * ATSC6p + (2.64078) * SssCH2.

Within this 2D model, the identified descriptors include Broto-Moreau autocorrelation—lag 7/weighted by I-state (ATS7s), Centered Broto-Moreau autocorrelation—lag 6/weighted by polarizabilities (ATSC6p), and the Sum of atom-type E-State: –CH2–(SssCH2). This model highlights the importance of a specific spatial distribution within this metabolite. At the same time, SssCH2 stands out as the only descriptor directly related to chemical groups. Moreover, as 3′ME5S, the molecule with the highest affinity, possess the smallest positive value. In this way, as SssCH2 the sum of the E-States for carbon atoms with two hydrogens attached and two single bonds to a nonhydrogen atoms, a lower positive E-states of the CH2 could enhance the interactions within the system[64]. Therefore, Metabolites interaction in the system are influenced by a particular spatial distribution, a wider negative charge repetition, and a lower positive E-State.

In summary, for Mordred, the optimal model highlighted the importance of descriptors like RNCG, MWC05, and ATSC6p, suggesting a wider distribution of negative charge enhances binding. PaDEL 3D emphasized the significance of spatial arrangement with descriptors TDB10m, RDF105p, and RDF115e. PaDEL 2D revealed the importance of specific spatial distribution, wider negative charge repetition, and lower positive E-State for metabolite interactions with GPER. In conclusion, our findings provide insights into molecular features crucial for GPER interactions, offering a foundation for further optimization and understanding of the underlying mechanisms.

## Conclusions

In conclusion, metabolites derived from (-)-Epicatechin, such as (-)-Epicatechin-3′-O-glucuronide (E3′G), (-)-Epicatechin-3′sulfate (E3′S), 3′-O-metyl-(-)-Epicatechin-5-sulfate (3′ME5S), 3′-O-metyl-(-)-Epicatechin-7-sulfate (3′ME7S), 5-(4′-hidroxyphenyl)-*γ*-verolactone-3′-sulfate (*γ*VL3′S), and 5-(4′-hidroxyphenyl)-*γ*-verolactone-3′-O-glucuronide (*γ*VL3′G), show significant potential as GPER ligands.

The literature suggests that the interaction of these metabolites with the GPER receptor may have important implications for cardiovascular health, including the activation of endothelial nitric oxide synthase (eNOS). Additionally, it has been shown that GPER activation by (-)-Epicatechin metabolites can improve endothelial function and reduce inflammation, which may contribute to the prevention of cardiovascular diseases.

The computational approaches used in the study, such as density functional theory (DFT) molecular docking, and molecular dynamics simulation, have provided detailed information on the physicochemical properties and binding affinity of (-)-Epicatechin metabolites with GPER. These approaches have expanded our understanding of the molecular interactions between the metabolites and the receptor, which may be crucial for the development of future therapeutic strategies focused on GPER.

Taken together, these findings underscore the importance of continuing to investigate the effects of (-)-Epicatechin metabolites on cardiovascular health, as well as the need to explore their therapeutic potential in the context of cardiovascular diseases. This emerging field of research offers exciting opportunities for the development of nutrition and pharmacology-based interventions that could have a significant impact on cardiovascular health.

## Supplementary Information

Below is the link to the electronic supplementary material.

Supplementary file1 (DOCX 228 KB)

## Data Availability

No datasets were generated or analysed during the current study.
