# Supplementary material for: (-)-Epicatechin metabolites as a GPER ligands: a theoretical perspective
Source: Mol Divers. 2024 Aug 17;29(3):2099–115. doi: 10.1007/s11030-024-10968-9 (PMC12081483; doi:10.1007/s11030-024-10968-9)
Supplement: Supplementary file 1 — Supplementary file1 (DOCX 228 KB) [file 11030_2024_10968_MOESM1_ESM.docx]

# Supplementary Material

# (-)-Epicatechin metabolites as a GPER ligands: a theoretical perspective.

Rodolfo Daniel Ávila-Avilés^1,2^, Erick Bahena-Culhuac^1,2^, and J. Manuel Hernández-Hernández *^1^

^1^ Laboratory of Epigenetics of Skeletal Muscle Regeneration, Department of Genetics and Molecular Biology, Centre for Research and Advanced Studies of IPN (CINVESTAV), Mexico City, Mexico.

^2^ Transdisciplinary Research for Drug Discovery, Sociedad Mexicana de Epigenética y Medicina Regenerativa A. C. (SMEYMER); Mexico City, Mexico.

*Corresponding author: [jose.hernandezh@cinvestav.mx](mailto:jose.hernandezh@cinvestav.mx)


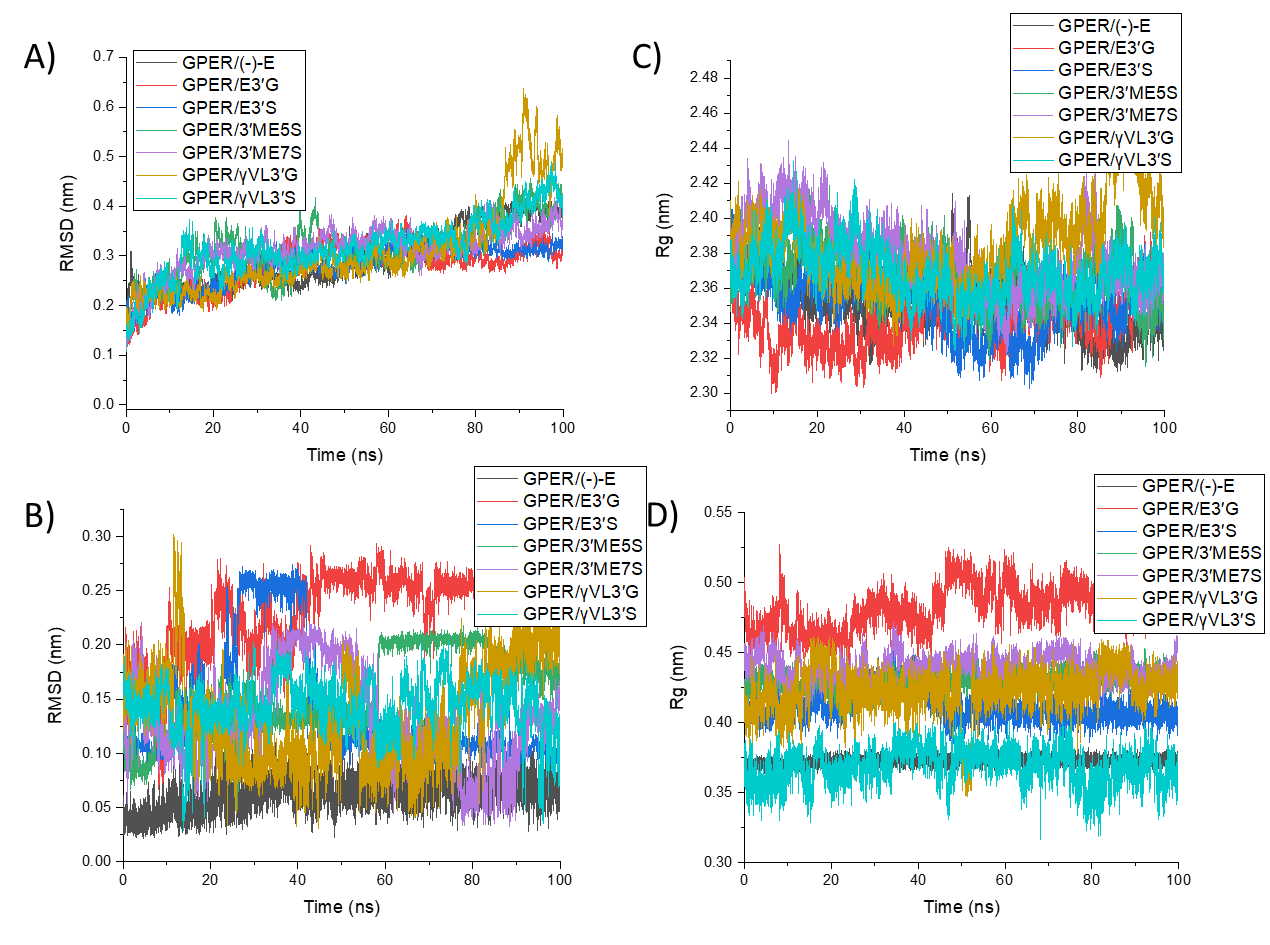


Figure S1. RMSD of GPER protein A), (-)-Epicatechin, and metabolites B): E3′G, E3′S, 3′ME5S, 3′ME7S, γVL3′G, and γVL3′S; and RG of GPER protein C), (-)-Epicatechin, and metabolites D): E3′G, E3′S, 3′ME5S, 3′ME7S, γVL3′G, and γVL3′S.


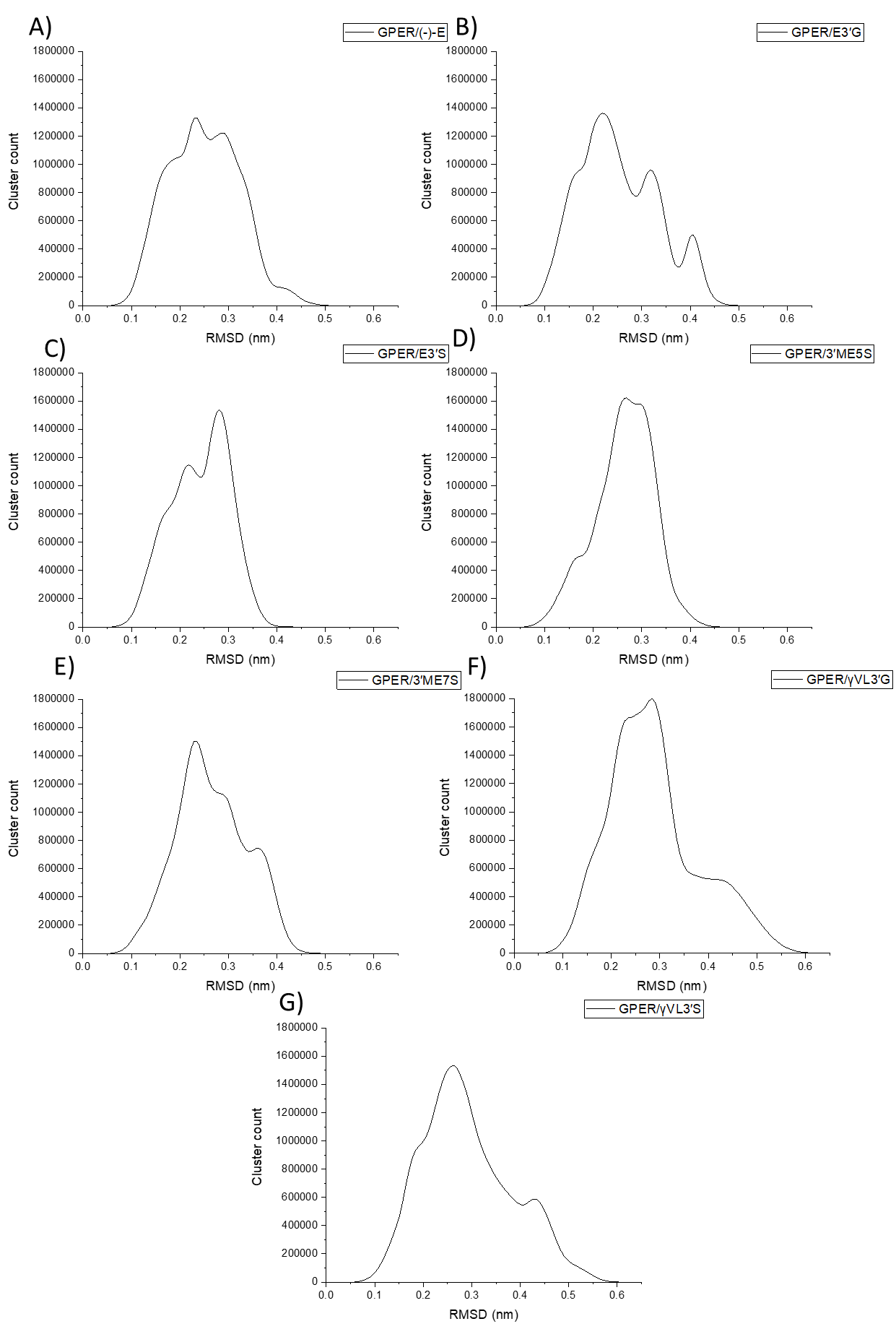


Figure S2. Cluster analysis distribution of GPER protein in complex with (-)-Epicatechin, and metabolites: E3′G, E3′S, 3′ME5S, 3′ME7S, γVL3′G, and γVL3′S


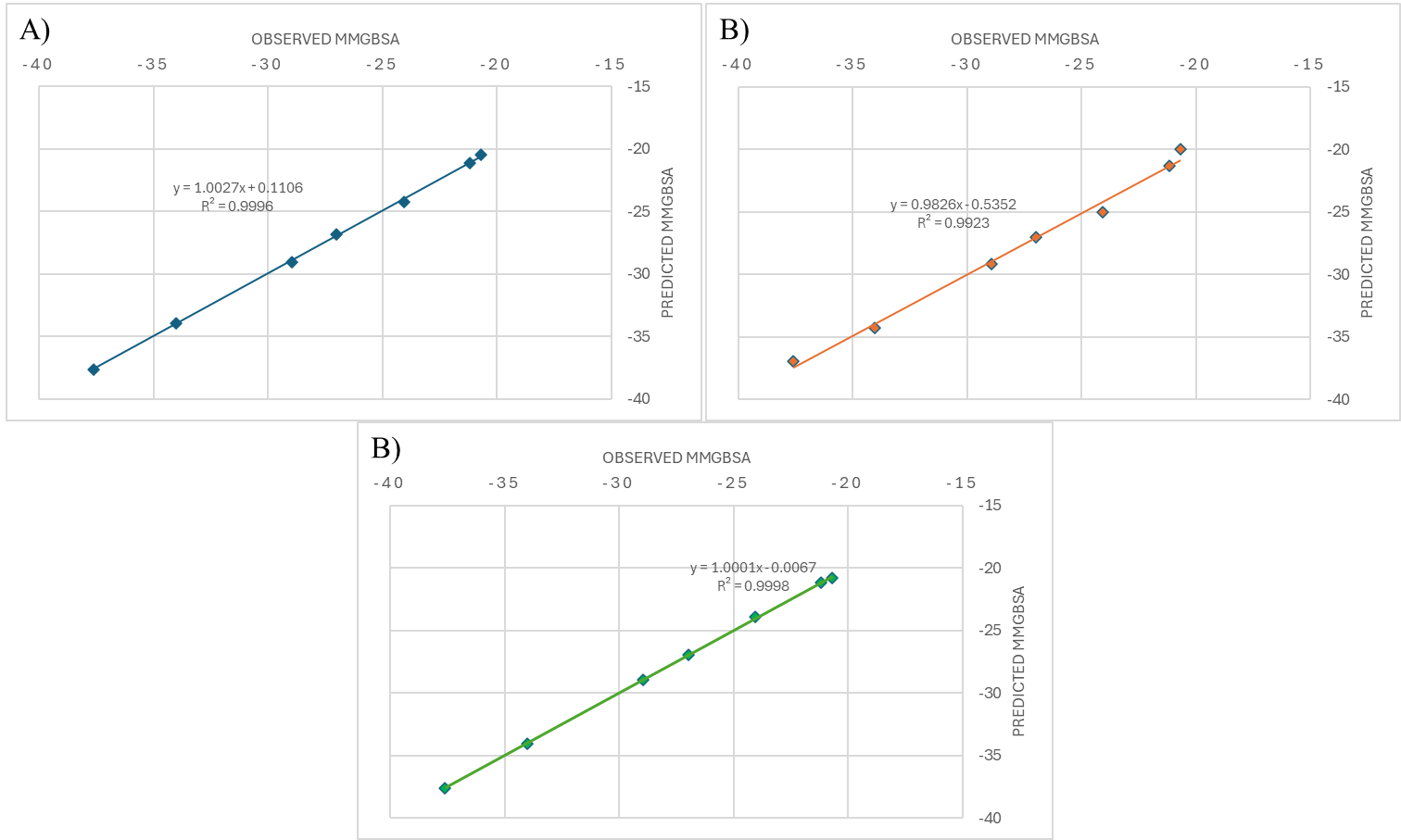


Figure S3. Graph of correlation between observed vs predicted MMGBSA based on the best model of A) Mordred, B) PaDEL 3D, and C) PaDEL 2D descriptors.

Supplementary Table 1. Values of Molecular Properties Within the Optimal Range for Absorption, Distribution, Metabolism, Excretion, and Toxicity (ADMET) of (-)-Epicatechin and metabolites

| Absorption | (-)-Epicatechin | | E3′G | | E3′S | | 3′ME5S | | 3′ME7S | | γVL3′S | | γVL3′G | |
| --- | --- | --- | --- | --- | --- | --- | --- | --- | --- | --- | --- | --- | --- | --- |
| Property | Value | Dec. | Value | Dec. | Value | Dec. | Value | Dec | Value | Dec. | Value | Dec. | Value | Dec. |
| Caco-2 Permeability | -6.04 | No | -6.32 | No | -6.34 | No | -6.04 | No | -6.27 | No | -5.60 | No | -5.88 | No |
| MDCK Permeability | -4.94 | No | -4.87 | No | -4.98 | No | -5.10 | No | -5.18 | No | -4.67 | No | -4.89 | No |
| PAMPA | 0.737 | No | 1 | No | 0.923 | No | 0.29 | Yes | 1 | No | 0.694 | No | 0.987 | No |
| Pgp-inhibitor | 0 | Yes | 0 | Yes | 0.001 | Yes | 0.03 | Yes | 0 | Yes | 0.19 | Yes | 0.001 | Yes |
| Pgp-substrate | 0.245 | Yes | 0.965 | No | 0.416 | No | 0.15 | Yes | 0.915 | No | 0.377 | No | 0.119 | Yes |
| HIA | 0.052 | Yes | 0.783 | No | 0.242 | Yes | 0.00 | Yes | 0.981 | No | 0.394 | No | 0.214 | Yes |
| F-20% | 0.865 | No | 0.871 | No | 0.9 | No | 0.41 | No | 0.738 | No | 0.929 | No | 0.05 | Yes |
| F-30% | 0.983 | No | 0.982 | No | 0.989 | No | 0.83 | No | 0.921 | No | 0.946 | No | 0.137 | Yes |
| F-50% | 0.998 | No | 0.999 | No | 0.998 | No | 0.96 | No | 0.998 | No | 0.893 | No | 0.554 | No |
| Distribution | (-)-Epicatechin | | E3′G | | E3′S | | 3′ME5S | | 3′ME7S | | γVL3′S | | γVL3′G | |
| Property | Value | Dec. | Value | Dec. | Value | Dec. | Value | Dec | Value | Dec. | Value | Dec. | Value | Dec. |
| Plasma Protein Binding | 92.78 | No | 74.65 | Yes | 93.36 | No | 94.3 | No | 67.06 | Yes | 64.25 | Yes | 69.51 | Yes |
| Volume Distribution | 0.059 | Yes | -0.33 | No | -0.40 | No | -0.2 | No | -0.43 | No | -0.83 | No | -0.69 | No |
| Blood-Brain Barrier Penetration | 0.09 | Yes | 0 | Yes | 0.004 | Yes | 0 | Yes | 0 | Yes | 0.006 | Yes | 0.004 | Yes |
| Fractiln Unboun in Plasms | 14.33 | Yes | 25.96 | Yes | 5.389 | Yes | 6.46 | Yes | 34.40 | Yes | 32.9 | Yes | 30.31 | Yes |
| OATP1B1 inhibitor | 0.338 | No | 0.001 | Yes | 0.075 | Yes | 0.99 | No | 0.019 | Yes | 0.55 | No | 0.213 | Yes |
| OATP1B3 inhibitor | 0.951 | No | 0.59 | No | 0.927 | No | 0.99 | No | 0.949 | No | 0.426 | No | 0.833 | No |
| BCRP inhibitor | 0.204 | Yes | 0.004 | Yes | 0.462 | No | 0.18 | Yes | 0.009 | Yes | 0.496 | No | 0.007 | Yes |
| MRP1 inhibitor | 0.919 | No | 0.995 | No | 0.979 | No | 0.92 | No | 0.995 | No | 0.767 | No | 0.981 | No |
| Metabolism | (-)-Epicatechin | | E3′G | | E3′S | | 3′ME5S | | 3′ME7S | | γVL3′S | | γVL3′G | |
| Property | Value | Dec. | Value | Dec. | Value | Dec. | Value | Dec | Value | Dec. | Value | Dec. | Value | Dec. |
| CYP1A2 inhibitor | 0 | Yes | 0 | Yes | 0 | Yes | 0 | Yes | 0 | Yes | 0 | Yes | 0 | Yes |
| CYP1A2 substrate | 0.065 | Yes | 0 | Yes | 0 | Yes | 0 | Yes | 0 | Yes | 0 | Yes | 0.141 | Yes |
| CYP2C19 inhibitor | 0 | Yes | 0 | Yes | 0 | Yes | 0 | Yes | 0 | Yes | 0 | Yes | 0 | Yes |
| CYP2C19 substrate | 0.001 | Yes | 0 | Yes | 0 | Yes | 0 | Yes | 0 | Yes | 0 | Yes | 0.672 | No |
| CYP2C9 inhibitor | 0 | Yes | 0 | No | 0 | Yes | 0 | Yes | 0 | Yes | 0 | Yes | 0 | Yes |
| CYP2C9 substrate | 0.876 | No | 0.999 | No | 1 | No | 0.94 | No | 0.983 | No | 0.804 | No | 0.819 | No |
| CYP2D6 inhibitor | 0 | Yes | 0 | Yes | 0 | Yes | 0 | Yes | 0 | Yes | 0 | Yes | 0 | Yes |
| CYP2D6 substrate | 0.8893 | No | 0.006 | Yes | 0 | Yes | 0 | Yes | 0 | Yes | 0 | Yes | 0 | Yes |
| CYP3A4 inhibitor | 0.072 | Yes | 0 | Yes | 0 | Yes | 0 | Yes | 0 | Yes | 0 | Yes | 0.002 | Yes |
| CYP3A4 substrate | 0 | Yes | 0 | Yes | 0 | Yes | 0 | Yes | 0 | Yes | 0 | Yes | 0.688 | No |
| CYP2B6 inhibitor | 0 | Yes | 0 | Yes | 0 | Yes | 0 | Yes | 0 | Yes | 0 | Yes | 0 | Yes |
| CYP2B6 substrate | 0 | Yes | 0 | Yes | 0 | Yes | 0 | Yes | 0 | Yes | 0 | Yes | 0.867 | No |
| CYP2C8 inhibitor | 0.049 | Yes | 0 | Yes | 0 | Yes | 0 | Yes | 0 | Yes | 0.006 | Yes | 0 | Yes |
| Human liver microsomal (HLM) stability | 0.029 | Yes | 0.001 | Yes | 0 | Yes | 0 | Yes | 0 | Yes | 0.208 | Yes | 0.459 | No |
| Excretion | (-)-Epicatechin | | E3′G | | E3′S | | 3′ME5S | | 3′ME7S | | γVL3′S | | γVL3′G | |
| Property | Value | Dec. | Value | Dec. | Value | Dec. | Value | Dec | Value | Dec. | Value | Dec. | Value | Dec. |
| CL plasma penetration | 14.92 | No | 1.852 | Yes | 6.109 | No | 6.05 | No | 1.83 | Yes | 4.554 | Yes | 0.821 | Yes |
| Half Life | 2.14 | No | 3.78 | No | 2.04 | No | 2.286 | No | 4.088 | No | 1.365 | No | 1.277 | No |
| Toxicity | (-)-Epicatechin | | E3′G | | E3′S | | 3′ME5S | | 3′ME7S | | γVL3′S | | γVL3′G | |
| Property | Value | Dec. | Value | Dec. | Value | Dec. | Value | Dec | Value | Dec. | Value | Dec. | Value | Dec. |
| hERG Blockers | 0.143 | Yes | 0.029 | Yes | 0.029 | Yes | 0.03 | Yes | 0.046 | Yes | 0.015 | Yes | 0.054 | Yes |
| hERG Blockers (10um) | 0.776 | No | 0.177 | Yes | 0.172 | Yes | 0.12 | Yes | 0.197 | Yes | 0.06 | Yes | 0.151 | Yes |
| Drug Induced Liver Injury | 0.177 | Yes | 0.408 | No | 0.864 | No | 0.84 | No | 0.677 | No | 0.987 | No | 0.915 | No |
| AMES Mutagenicty | 0.525 | No | 0.288 | Yes | 0.204 | Yes | 0.31 | No | 0.344 | No | 0.698 | No | 0.284 | Yes |
| Rat Oral Acute Toxicity | 0.5 | No | 0.457 | No | 0.221 | Yes | 0.16 | Yes | 0.407 | No | 0.265 | Yes | 0.09 | Yes |
| FDAAMDD | 0.759 | No | 0.616 | No | 0.931 | No | 0.90 | No | 0.431 | No | 0.926 | No | 0.158 | Yes |
| Skin Sensitization | 0.876 | No | 0.062 | Yes | 0.734 | No | 0.83 | No | 0.04 | Yes | 0.998 | No | 0.897 | No |
| Carcinogenicity | 0.377 | No | 0.084 | Yes | 0.227 | Yes | 0.28 | Yes | 0.092 | Yes | 0.482 | No | 0.371 | No |
| Eye Corrosion | 0.005 | Yes | 0 | Yes | 0.01 | Yes | 0.14 | Yes | 0 | Yes | 0.884 | No | 0.029 | Yes |
| Eye Irritation | 0.97 | No | 0.174 | Yes | 0.826 | No | 0.95 | No | 0.06 | Yes | 0.89 | No | 0.461 | No |
| Respiratory | 0.532 | No | 0.212 | Yes | 0.679 | No | 0.63 | No | 0.245 | Yes | 0.433 | No | 0.188 | Yes |
| Human Hepatotoxicty | 0.5 | No | 0.503 | No | 0.587 | No | 0.77 | No | 0.555 | No | 0.77 | No | 0.576 | No |
| Drug-induced Nephrotoxicity | 0.054 | Yes | 0.286 | Yes | 0.139 | Yes | 0.21 | Yes | 0.564 | No | 0.9 | No | 0.82 | No |
| Ototoxicity | 0.749 | No | 0.968 | No | 0.561 | No | 0.58 | No | 0.979 | No | 0.232 | Yes | 0.676 | No |
| Hematotoxicity | 0.032 | Yes | 0.024 | Yes | 0.047 | Yes | 0.09 | Yes | 0.058 | Yes | 0.188 | Yes | 0.363 | No |
| Genotoxicity | 0.836 | No | 0.797 | No | 0.98 | No | 0.96 | No | 0.388 | No | 0.996 | No | 0.096 | Yes |
| RPMI-8226 Immunitoxicity | 0.024 | Yes | 0.058 | Yes | 0.03 | Yes | 0.02 | Yes | 0.071 | Yes | 0.036 | Yes | 0.046 | Yes |
| A549 Cytotoxicity | 0.697 | No | 0.06 | Yes | 0.025 |  | 0.01 | Yes | 0.067 | Yes | 0.015 | Yes | 0.011 | Yes |
| Hek293 Cytotoxicity | 0.689 | No | 0.556 | No | 0.502 | No | 0.273 | Yes | 0.474 | No | 0.054 | Yes | 0.074 | Yes |
| Drug Induced Neurotoxicity | 0.066 | Yes | 0.056 | Yes | 0.009 | Yes | 0.012 | Yes | 0.093 | Yes | 0.195 | Yes | 0.685 | No |
|  |  |  |  |  |  |  |  |  |  |  |  |  |  |  |
